# Modified completely intrafascial radical cysprostatectomy for bladder cancer: a single-center, blinded, controlled study

**DOI:** 10.1186/s12885-021-08568-z

**Published:** 2021-08-03

**Authors:** Xiao Wang, Jia Guo, Lei Wang, Min Wang, Xiaodong Weng, Hui Chen, Xiuheng Liu

**Affiliations:** Department of Urology, Renmin Hospital of Wuhan University, 238 Jiefang Road, Wuhan, 430060 P.R. China

**Keywords:** Radical cysprostatectomy, Intrafascial technique, Continence, Erectile function

## Abstract

**Background:**

We have proposed a modified, completely intrafascial radical cysprostatectomy (RC) to treat bladder cancer patients with the aim of preserving the patients’ post-surgical urinary control and erectile function. This study aimed to evaluate the oncological and functional outcomes of this innovation relatively to that with the conventional technique.

**Methods:**

A retrospective, single-center, blinded, and controlled study was conducted using the medical data of patients since the past 5 years from the hospital database. A total of 44 patients were included, including 20 who received complete intrafascial cysprostatectomy and 24 who received conventional interfascial surgeries. The patients’ continent and sexual information of 1-year follow-up after the surgery were extracted. The oncological and functional outcomes of the 2 groups were compared and analyzed.

**Results:**

The demographics parameters of the 2 groups showed no significant difference. The results of follow-up of the oncological outcomes did not reveal any significant difference between the completely intrafascial group and the conventional interfascial group in terms of the positive surgical margins, local recurrences, and distant metastasis. Patients following neobladder diversion in the intrafascial group showed a faster recovery of the urinary control, with a 76.9% (10/13) daytime continent rate at 3-month, as well as 46.2% (6/13) and 58.3% (7/12) nighttime continent rates at 3-month and 6-month, respectively. Regarding the sexual functions, our results revealed significant advantages in favor of completely intrafascial technique on the post-surgical International Index of Erectile Function (IIEF)-5 score at 3-, 9-, and 12-month follow-up relative to that with the conventional interfascial process. Thus, the IIEF score of patients in the intrafascial group was 11.4 ± 3.5 at 3-month, 14.1 ± 3.6 at 9-month, and 15.2 ± 3.8 at 12-month follow-up after the cystectomy, which was significantly greater than that of the patients in the control group.

**Conclusions:**

Our novel data illustrated that the modified completely intrafascial technique could result in a better sexual function and faster continence recovery for patients following RC, without any compromise in the cancer control. Thus, this technique could be considered as an alternative extirpative technique for bladder cancer treatment in a clinical setting.

## Introduction

Radical cysprostatectomy (RC) is recommended as the standard treatment for muscle-invasive bladder cancer (MIBC) or refractory high risk non-MIBC (NMIBC) [1]. However, this process has often been associated with significant morbidities, which may be bothersome and interfere with the quality of life (QoL) [2, 3]. In recent times, several preservation techniques have been applied for younger and pre-surgically potent patients in order to improve their sexual functions and voiding functions, including prostate/prostatic capsule-sparing technique, seminal-sparing technique, and nerve-sparing technique [4, 5]. However, controversy has gone on that some of the preservation techniques may compromise cancer control [6].

Intrafascial technique has been widely utilized in prostatectomy, characterized by the medial dissection to the prostatic fascia, thereby maximally preserving the periprostatic nerve fibers internal to the prostatic fascia [7–9]. Some surgeons also performed intrafascial technique in RC so as to preserve patients’ post-operative erectile functions (EF). Anatomical studies have illustrated that autonomic nerves splitting from the pelvic plexus traveled lateral to the seminal vesicles [10]. A recent study through immunohistochemical staining and computed 3-dimensional reconstruction indicated that the mean distance between the nerves to the seminal vesicles was 1.68, 1.50, and 1.76 mm at the tip, middle, and base, respectively [11]. Thus, we modified our maneuver by dissecting the retrovesical plane and removing the seminal vesicles internal to the seminal vesicle fascia (SVF) perspective to preserve the abutting nerves. Along with the intrafascial process in the dissection of the peri-protatic tissues, we thus proposed the approach of “completely intrafascial” nerve-sparing cysprostatectomy. In the present study, we retrospectively assessed the oncological and functional outcomes of patients undergoing completely intrafascial RC in comparison with the conventional interfascial technique. We sought to understand the differences in approaches to cancer control, voiding, and sexual function between these 2 patient populations in order to determine the potential of the proposed approaches as alternative extirpative techniques for bladder cancer treatment.

## Methods

### Study design

This was a retrospective, single-center, blinded and controlled study based on the evaluation of patients’ information in the past 5 years. From January 2015 to October 2019, more than 150 RCs were performed at our center, among which approximately one-third cases were subjected to nerve-sparing techniques. We reviewed all the patient cases who received RCs and, finally, 44 patients were included in our analysis, of which 20 underwent the completely intrafascial surgery (completely intrafascial group, interfered group) and 24 patients underwent the conventional interfascial surgery (conventional interfascial group, control group). The patient inclusion criteria involved male sex; pre-operative normal sexual function (IIEF-5 score > 21) or mild erectile dysfunction (IIEF-5 score = 12–21); taking nerve-sparing techniques, including either completely intrafascial technique or conventional nerve-sparing technique; bladder tumor pathology of urothelial carcinoma; and the availability of follow-up and relevant information after the surgery. Exclusion criteria included: 1) surgery videos were missed and the specific nerve-sparing techniques were uncertain; 2) the surgeries were not performed by XHL; 3) deficiency of the patients’ pre-operative information; 4) suspicious prostate cancer according to pathologic PSA/DRE/TURS. There was no limitation on the patients’ age or the tumor stage and cases of both opened and laparoscopic surgeries were included. Consent of sexual preservation was pre-surgically acquired from all included patients, although the detailed maneuver of nerve-sparing techniques were blinded for patients. The patients were followed-up at 1, 3, 6, 9, and 12 months in the first year, every 6 months in the next 2 years, and then yearly by the urologists and nurses. The patients’ functional outcomes of 1-year follow-up after the surgery were extracted and analyzed in the present study.

### Surgical technique

**Fig. 1:**
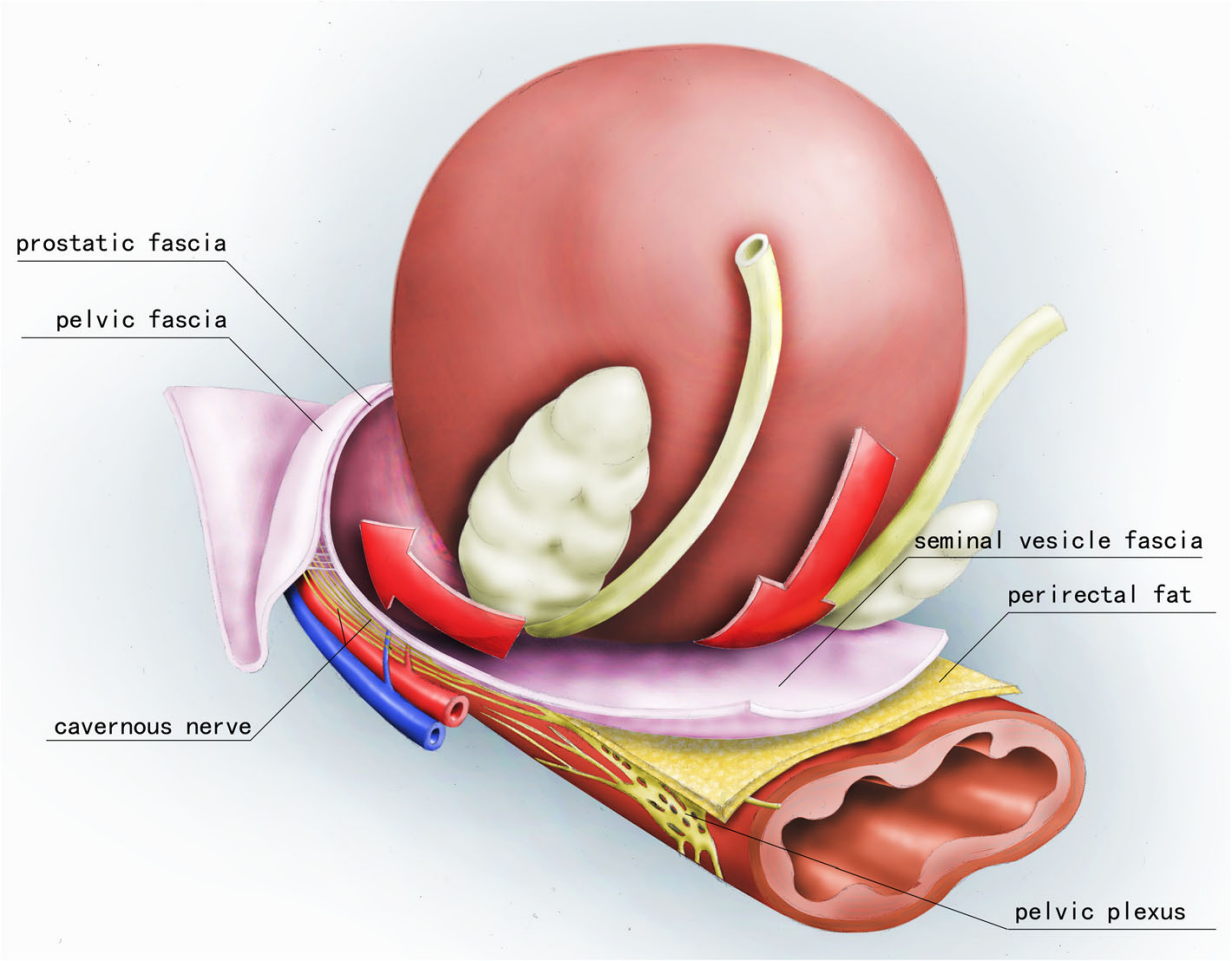
Schematics of completely intrafascial modification to approach the intrafascial plane. Deferential ampullas were searched along with the deferent duct caudally. The seminal vesicles were found lateral to the deferential ampullas and mobilized with blunt dissection. All the dissections were performed in the plane anterior/internal to the seminal vesicle fascia. At the level of the prostatic base, Denonvilliers fascia was exposed and dropped down away from the posterior prostatic capsule without entering the plane of perirectal fat. This avascular intrafascial plane anterior to Denonvilliers fascia was extended down to the apex of the prostate and could be developed between the prostatic capsule and peri-prostatic fascia from post-lateral to the dorsal surface of the prostate

**Fig. 2:**
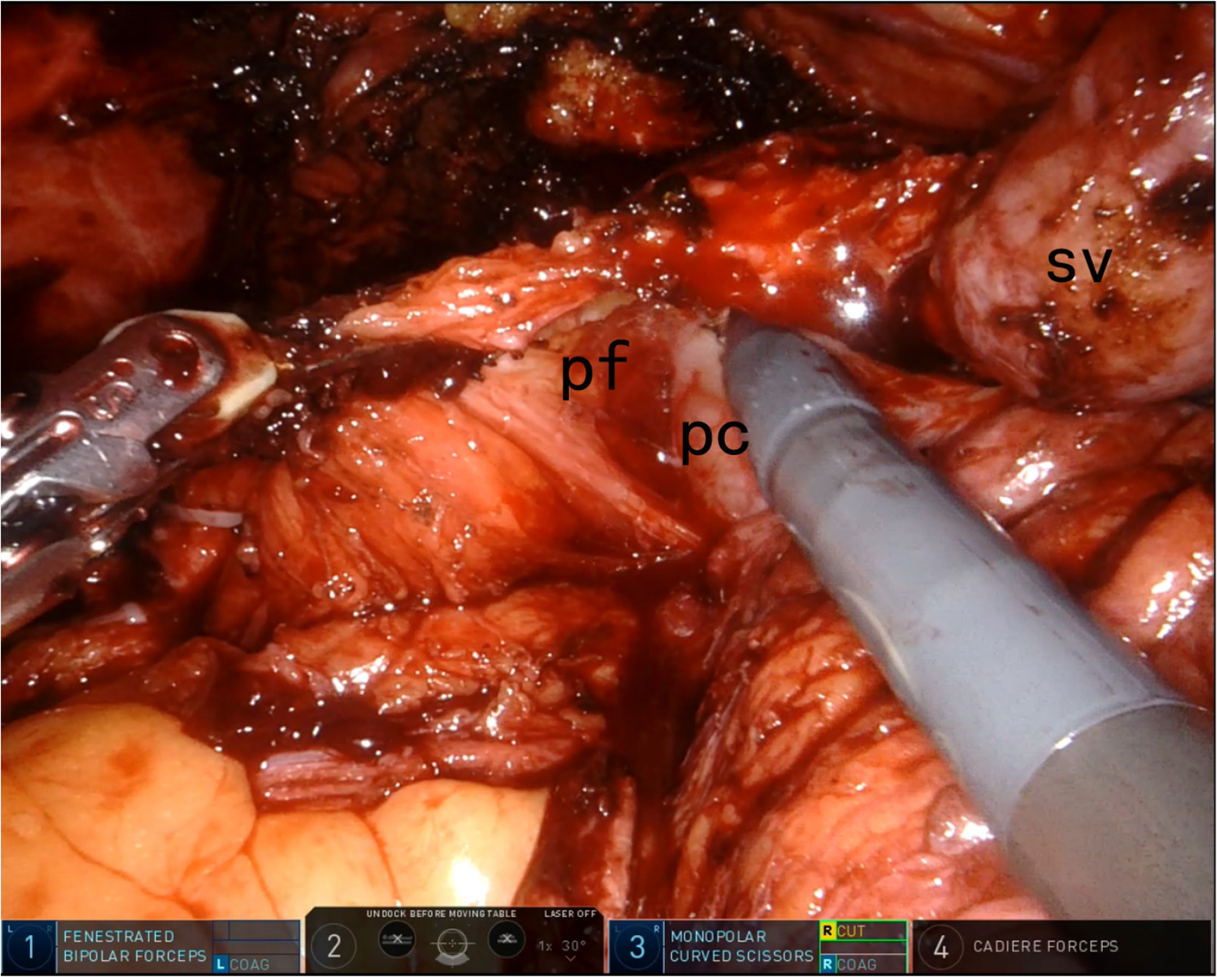
Laparoscopic photo depicting the intrafascial plane. The seminal vesicles were mobilized. The avascular intrafascial plane was entered by recognizing the smooth and reflecting prostatic capsule. pc = prostatic capsule; pf = prostatic fascia; sv = seminal vesicle

All surgeries were performed by the same surgeon (XHL). From 2017, we modified the conventional nerve-sparing process and proposed a completely intrafascial technique approach. The main difference with respect to the standard nerve-sparing cystectomy was that the peritoneum was incised in the transverse fashion along with the deferent duct cleavage anterior to the cul-de-sac. Blunt dissection could be performed to mobilize the seminal vesicles. As nerves from the pelvic plexus adjoined the seminal vesicles, gentle maneuvers and the use of a cold apparatus were thus suggested. All the dissections were performed in the plane anterior/internal to the SVF. At the level of the prostatic base, Denonvilliers fascia was exposed. We did not incise the Denonvilliers fascia to enter the plane of the perirectal fat. Sharp dissection was employed to drop down Denonvilliers fascia away from the posterior prostatic capsule. This plane anterior to the Denonvilliers fascia was extended caudally to the apex of the prostate. Then we performed intrafascial dissection of the prostate in reference to the “superveil” technique [12]. Figure 1 depicts the schematics of completely intrafascial modification, while Fig. 2 presents the laparoscopic photo of the intrafascial plane.

In the control group, the conventional interfascial technique was performed. Unlike with the completely intrafascial process, the posterior peritoneal reflection was incised at the cul-de-sac to enter the perirectal plane. The DVC was ligated and the pelvic fascia was incised. NVBs were preserved dorsolaterally to the prostate through ligation and division of the vesical and prostatic pedicles.

The videos of the surgery were reviewed to reconfirm the preservation techniques. The main identifications of nerve-sparing procedures through videos to distinguish the intra- or interfascial techniques involved the incision position of peritoneum, whether to incise the Denonvilliers fascia to enter the plane of the perirectal fat, whether to incise pelvic fascia and ligate the DVC, and discrimination of the intrafascial plane between prostatic capsule and prostatic fascia.

### Outcomes and follow-up

The baseline assessments included patient demographic characteristics, tumor stage, and diversion type. The pre-surgical IIEF-5 scores were recorded. The examination of the perioperative characteristics included the estimated blood loss, operative duration, and positive surgical margins (PSMs). Local recurrence and metastatic tumor were estimated through imaging examinations. Local recurrence was defined as any urothelial cancer recurrence below the iliac bifurcation in pelvic soft tissues. The functional outcomes followed-up included voiding and sexual functions as well as the recovery duration. Patients following neobladder diversion acquiring no urinary loss and using no pads or 1 safety pad were identified as continence at daytime or night-time. The IIEF-5 scores were obtained via interview and > 12 (mild erectile dysfunction or normal sexual function) was defined as post-surgical potency. Phosphodiesterase type 5 inhibitors (PDE5-I; sildenafil citrate, 50 mg) on demand were prescribed if the patients suffered from severe erectile dysfunction (IIEF-5 score < 8) or reported unsatisfied intercourse after the first month of follow-up.

### Statistical analysis

The data was processed using the SPSS 17.0 software for Windows. Statistical analysis of continuous variable means was performed using Student *t*-tests after normality test and variance equality test, while the categorical data was analyzed by Chi-square test. Survival analysis was employed to compare the recovery of potency between the complete intrafascial group and the conventional interfascial group by using the Kaplan–Meier methods. Logistic model was built to adjust the influence of confounding factors. The probability of type I error was considered at 0.05, and all tests were 2-sided.

## Results

From January 2015 to October 2019, more than 150 patients underwent RCs for bladder cancer at our center and, finally, 44 male patients met all the inclusion criteria and were included in the present study. Of these, 20 patients received completely intrafascial RCs and 24 patients underwent the conventional interfascial RCs. In the control group, 4 patients in the completely intrafascial group were transformed to the conventional interfascial procedure from completely intrafascial RCs due to intraoperative suspected seminal vesicle invasion (*n* = 3) or severe conglutination in the intrafascial plane (*n* = 1). During the first year follow-up, 1 patient was reported dead at 9 months after the surgery and 2 patients were missed at the 6th and 9th month follow-up in the completely intrafascial group. Meanwhile, 2 patients were missed at the 6th and 9th month of follow-up after RCs in the control group. Table 1 shows the demographics and the perioperative and pre-operative parameters of 44 included patients. The demographics parameters of the 2 groups showed no significant difference, including with respect to the mean age, BMI, smoking proportion, hypertension rate, and diabetes rate. Most surgeries were performed by laparoscopy in both the groups. One patient in the completely intrafascial group was selected for open surgery because of the history of abdominal surgery, while 1 patient in the control group was transformed to open surgery due to intraoperative rectal injury. Moreover, 13 (65.0%) and 19 (79.2%) patients received orthotopic neobladder in the completely intrafascial group and the conventional interfascial group, respectively. No statistically significant differences were noted in terms of the operation duration and blood loss between the 2 processes. The patients’ pre-operative sexual functions were found to be consistent between the 2 groups. Specifically, the mean pre-operative IIEF-5 scores in the completely intrafascial group was 16.3, which showed a slight non-significant inferiority when compared with that of 17.1 in the control group. In addition, 3 (15.0%) patients showed normal sexual function and 17 (85.0%) showed mild erectile dysfunction, who underwent completely intrafascial RCs. On the other hand, in the control group, 7 (29.2%) and 17 (70.8%) patients showed normal sexual function and mild erectile dysfunction, respectively.

**Table 1:** Demographics, perioperative, and pre-operative parameters of 44 study patients

	Completely intrafascial group	Conventional interfascial group	*P* value
No. pts.	20	24	–
Mean *mon* follow-up (SD)	15.7 (4.4)	22.2 (7.5)	**0.001**
Mean *yrs* age (SD)	66.2 (9.5)	61.8 (9.8)	0.149
Mean *kg/m*^*2*^ BMI (SD)	24.7 (3.7)	24.3 (4.4)	0.749
No. smoking (%)	8 (40.0)	11 (45.8)	0.697
No. hypertension (%)	8 (40.0)	6 (25.0)	0.287
No. diabetes (%)	7 (35.0)	9 (37.5)	0.864
No. NAC (%)	6 (30.0)	7 (29.2)	0.952
No. surgery type (%)			0.895
open	1 (5.0)	1 (4.2)	
aparoscopic	19 (95.0)	21 (95.8)	
No. diversion type (%)			0.544
orthotopic neobladder	13 (65.0)	19 (79.2)	
ileal conduit	5 (25.0)	4 (16.7)	
ureterocutaneostomy	2 (10.0)	1 (4.2)	
Mean *mins* operation duration (SD)	339.4 (46.1)	343.9 (40.5)	0.731
Mean *ml* estimated blood loss (SD)	518.3 (383.9)	565.8 (382.3)	0.684
Mean pre-operative IIEF-5 score (SD)	16.3 (3.8)	17.1 (4.5)	0.539
No. pre-operative sexual function (%)			0.264
normal sexual function	3 (15.0)	7 (29.2)	
mild erectile dysfunction	17 (85.0)	17 (70.8)	
No. dead pts. during follow-up (%)	1 (5.0)	0 (0)	0.268
No. missing pts. during follow-up (%)	2 (10.0)	2 (8.3)	0.848

Postsurgical complications were recorded and graded using Clavien classification system. We considered those that occurred up to 30 days after surgeries as early complications, and that occurred later than 30 days as late complications. As shown in Table 2, most complications were early complications and graded as Clavien I to III. No significant difference of complication incidence could be noted between the completely intrafascial group and the conventional interfascial group.

**Table 2:** Early and late surgical complications with Clavien grading

Complication	Completely intrafascial group (*n* = 20) No. (%)	Conventional interfascial group (*n* = 24) No. (%)	Clavien grade
Early complications (0-30d)			
Wound infection	0 (0)	1 (4.2)	I
Paralytic ileus	5 (25.0)	6 (25.0)	I
Lymphorrhea	6 (30.0)	4 (16.7)	I
Pyrexia	3 (15.0)	2 (8.3)	I
Anaemia	2 (10.0)	1 (4.2)	II
Pneumonia	1 (5.0)	3 (12.5)	II
Psychosis	1 (5.0)	0 (0)	II
Lymphocele	2 (10.0)	1 (4.2)	IIIa
Urinary leakage	3 (15.0)	2 (8.3)	IIIa
Obstructive ileus	1 (5.0)	1 (4.2)	IIIb
Deep vein thrombosis	0 (0)	1 (4.2)	IVa
Late complications (>30d)			
Pyelonephritis	2 (10.0)	1 (4.2)	II
Hydronephrosis	2 (10.0)	1 (4.2)	IIIa
Increased urine residual	5 (25.0)	4 (16.7)	IIIa
Orthotopic neobladder calculi	0 (0)	1 (4.2)	IIIb
Ureteroileal stricture	1 (5.0)	3 (12.5)	IIIb
Parastomal hernia	0 (0)	1 (4.2)	IIIb
Incisional hernia	0 (0)	1 (4.2)	IIIb

### Oncological outcomes

Overall, the results of pre-operative and followed-up oncological outcomes did not demonstrate any significant difference between the completely intrafascial group and the conventional interfascial group, although the follow-up duration of these 2 groups varied significantly. As shown in Table 3, in the interfered group, 12 (60.0%) patients had T2 stage cancer and 5 (25.0%) had T3 stage cancer, while the proportion of T2 and T3 stages were 17 (70.8%) and 4 (16.7%), respectively, in the control group. This result indicated that most patients included in this study were bothered with the clinical MIBC. The oncological results suggested a certain difference between the pathological stage and the clinical stage. In detail, 3 (15.0%) and 5 (20.8%) patients were recorded with pathological T4 disease in the completely intrafascial group and the conventional interfascial group, respectively. In addition, 6 cases (30.0%) were reported to be pN+ in the interfered group, while the respective proportion in the control group was 8 cases (33.3%). Chi-square test failed to detect any statistical differences in terms of the pN+ rates. The 2 surgical procedures demonstrated approximate PSMs rates, and the most frequently reported PSM sites were perivesical soft tissues. In the completely intrafascial group, 4 (20.0%) patients had local recurrences and another 4 (20.0%) had distant metastasis. On the other hand, local recurrences and metastatic diseases were recorded in 5 (20.8%) and 7 (29.2%) patients, respectively, in the conventional interfascial group.

**Table 3:** Pre-operative and followed-up oncological characteristics of 44 study patients

	Completely intrafascial group	Conventional interfascial group	*P* value
No. clinical T stage (%)			0.735
T1	3 (15.0)	3 (12.5)	
T2	12 (60.0)	17 (70.8)	
T3	5 (25.0)	4 (16.7)	
No. clinical N stage (%)			0.261
N0	16 (80.0)	22 (91.7)	
N+	4 (20.0)	2 (8.3)	
No. pathological T stage (%)			0.785
T0	1 (5.0)	2 (8.3)	
Ta/Tis	0 (0)	0 (0)	
T1	1 (5.0)	0 (0)	
T2	7 (35.0)	9 (37.5)	
T3	8 (40.0)	8 (33.3)	
T4	3 (15.0)	5 (20.8)	
No. pathological N stage (%)			0.813
N0	14 (70.0)	16 (66.7)	
N1	6 (30.0)	8 (33.3)	
No. positive surgical margins (%)	5 (25.0)	3 (12.5)	0.284
No. incidental prostate cancer (%)	2 (10.0)	1 (4.2)	0.445
No. local recurrence (%)	2 (10.0)	3 (12.5)	0.795
No. metastatic disease (%)	2 (10.0)	7 (29.2)	0.117

### Functional outcomes

**Fig. 3:**
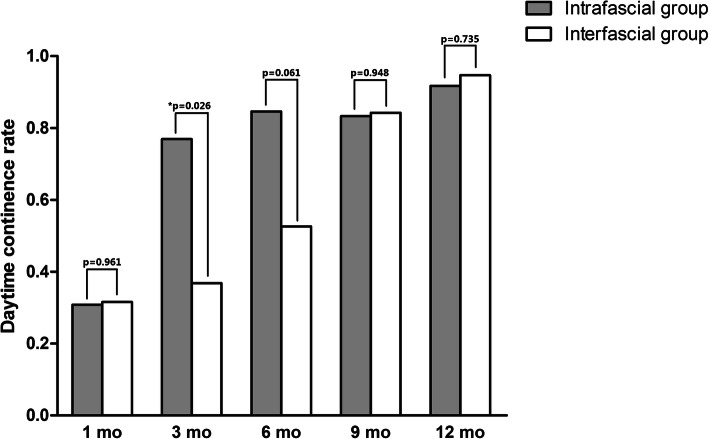
Comparison between completely intrafascial and conventionally interfascial groups regarding patients’ post-surgical daytime continence. Over 90% of the patients showed daytime continence at 1-year follow-up after the surgery in both the groups. The statistical difference could be tested only at 3-month follow-up for daytime continence rate, with a 76.9% (10/13) rate in the completely intrafascial group and a 36.8% (7/19) rate in the conventional interfascial group

**Fig. 4:**
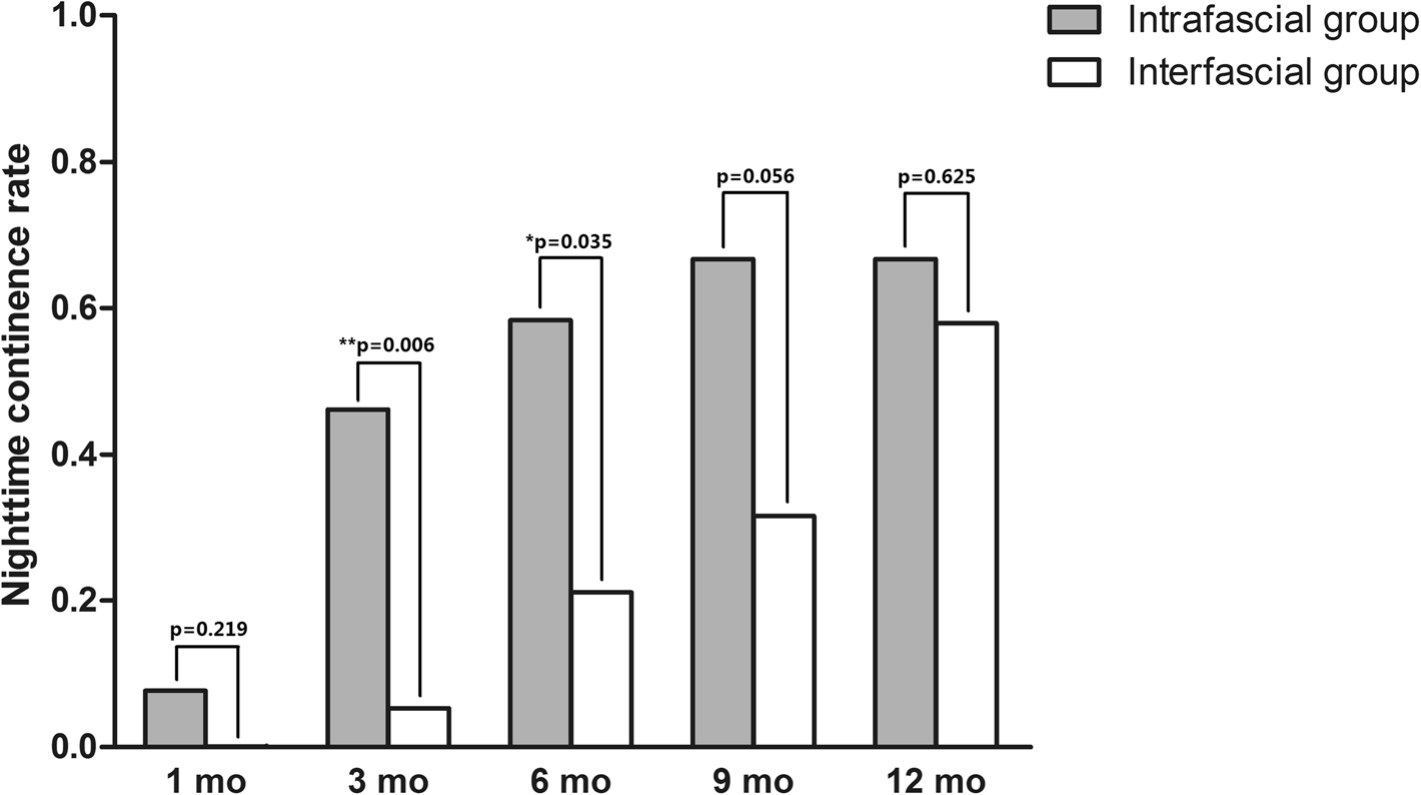
Comparison between completely intrafascial and conventionally interfascial groups regarding patients’ post-surgical night-time continence. Patients followed-up after completely intrafascial surgery showed better night-time urinary control relative to those after conventional procedure at the 3-month and 6-month follow-up. 46.2% (6/13) and 58.3% (7/12) patients in the inferred group reported continence at the night-time at 3-month and 6-month follow-up, respectively, which were significantly higher than those of the control group, in which only 5.3% (1/19) and 21.1% (4/19) patients were continent

Over 90% of the patients showed daytime continence at 1-year follow-up after the surgery in both the groups, although the comparison demonstrated no significant difference (Table 4; Fig. 3). The statistical difference could be tested only at 3-month follow-up for daytime continence rate, with a 76.9% (10/13) rate in the completely intrafascial group and a 36.8% (7/19) rate in the conventional interfascial group. Figure 4 demonstrated the comparisons in the night-time continence rates between the 2 groups, which indicates that patients followed-up after completely intrafascial surgery showed better night-time urinary control relative to those after conventional procedure at the 3-month and 6-month follow-up. In detail, 46.2% (6/13) and 58.3% (7/12) patients in the inferred group reported continence at the night-time at 3-month and 6-month follow-up, respectively. The rates were significantly higher than those of the control group, in which only 5.3% (1/19) and 21.1% (4/19) patients were continent. Moreover, 66.7% (8/12) patients underwent intrafascial cystectomy and 57.9% (11/19) patients, after interfascial surgery, had a night-time continence at 12-month, albeit the difference was insignificant.

**Table 4:** Comparison between completely intrafascial group and conventional interfascial group for continence

	Completely intrafascial group	Conventional interfascial group	*P* value
No. daytime continence (%)			
1 mo	4 (30.8)	6 (31.6)	0.961
3 mo	10 (76.9)	7 (36.8)	**0.026**
6 mo	11 (84.6)	10 (52.6)	0.061
9 mo	10 (83.3)	16 (84.2)	0.948
12 mo	11 (91.7)	18 (94.7)	0.735
No. nighttime continence (%)			
1 mo	1 (7.7)	0 (0)	0.219
3 mo	6 (46.2)	1 (5.3)	**0.006**
6 mo	7 (58.3)	4 (21.1)	**0.035**
9 mo	8 (66.7)	6 (31.6)	0.056
12 mo	8 (66.7)	11 (57.9)	0.625

**Fig. 5:**
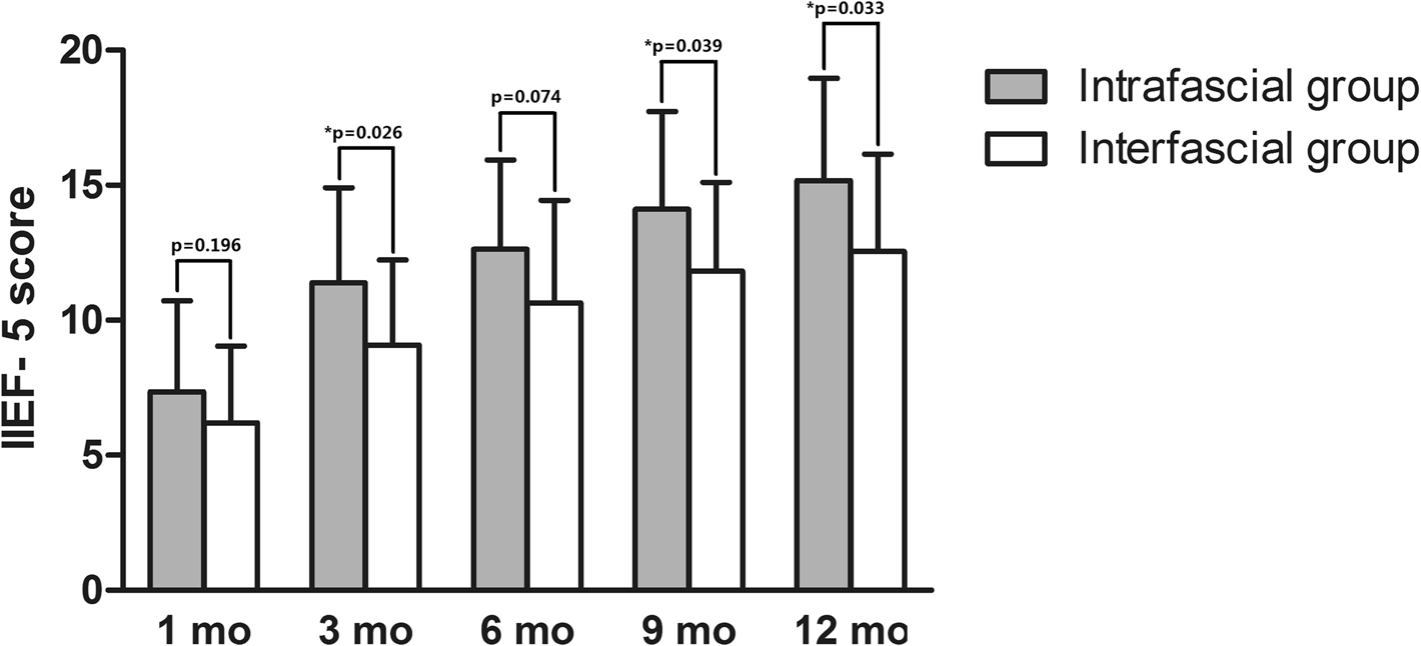
Comparison between completely intrafascial and conventionally interfascial groups regarding patients’ post-surgical IIEF-5 score. This figure demonstrated significant advantages in favor of completely intrafascial techniques on post-surgical IIEF-5 scores at 3-, 9-, and 12-month follow-up when compared with the conventional interfascial process. The IIEF score of patients in the inferred group was 11.4 ± 3.5 at 3-month, 14.1 ± 3.6 at 9-month, and 15.2 ± 3.8 at 12-month after cystectomy, which was significantly higher than that of patients in the control group with a *p* value of 0.026, 0.039, and 0.033, respectively

**Fig. 6:**
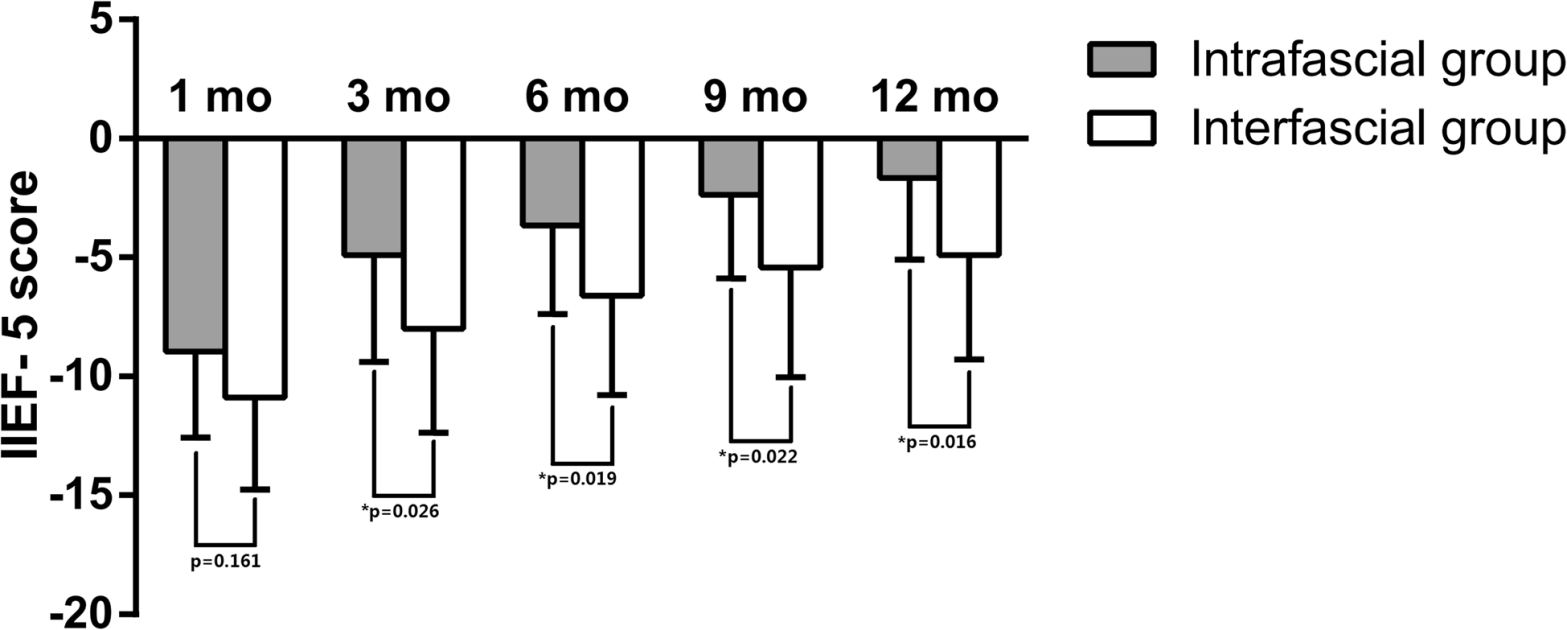
Comparison between completely intrafascial and conventionally interfascial groups regarding post-surgical IIEF-5 score change from baseline. This figure showed the changes in the IIEF-5 score from that at the baseline, only the data at 1-month failed to show any significant difference between the 2 groups. Mean change of IIEF-5 score from pre-surgical baseline in the inferred group was − 4.9 ± 4.5 at 3-month, − 3.7 ± 3.7 at 6-month, − 2.4 ± 3.5 at 9-month, and − 1.7 ± 3.4 at 12-month after cystectomy, which was significantly higher than that of patients in the control group

**Fig. 7:**
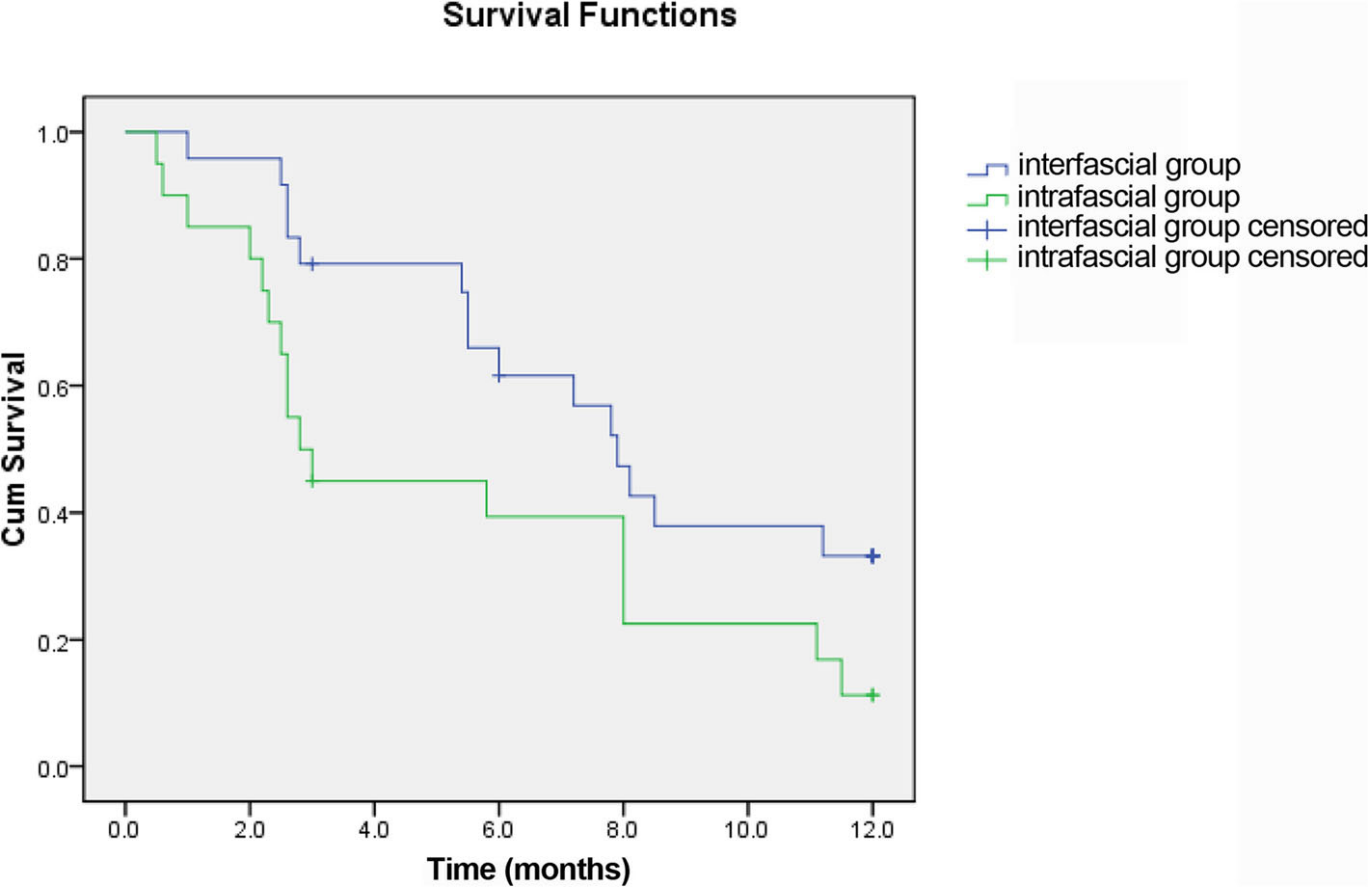
Survival plot indicating the recovery of potency between completely intrafascial group and conventionally interfascial group. Survival analysis was conducted to compare the recovery of potency between the 2 groups. The end-point event was set as patients achieving normal sexual function (IIEF-5 score > 21) or mild erectile dysfunction (IIEF-5 score = 12–21) after the surgery. This figure showed that patients in the intrafascial group had faster recovery duration with a median survival time of 2.8 months when compared with that in the control group with a median survival time of 7.9 months. The Kaplan–Meier method was used, and statistically significant difference was set at *p* = 0.049

We prescribed on demand PDE5-Is to patients who suffered from severe erectile dysfunction (IIEF-5 score < 8) or had unsatisfied intercourse after the first month of surgery. As shown in Table 5, no statistically significant difference was observed between the 2 groups in terms of the adoption of PDE5-Is. With regard to the sexual function, our results demonstrated significant advantages in favor of completely intrafascial techniques on post-surgical IIEF-5 scores at 3-, 9-, and 12-month follow-up when compared with the conventional interfascial process (Table 5; Fig. 5). In detail, the IIEF score of patients in the inferred group was 11.4 ± 3.5 at 3-month, 14.1 ± 3.6 at 9-month, and 15.2 ± 3.8 at 12-month after cystectomy, which was significantly higher than that of patients in the control group (*p* = 0.026, 0.039, and 0.033, respectively). Regarding the changes in the IIEF-5 score from that at the baseline, only the data at 1-month failed to show any significant difference between the 2 groups (Table 5; Fig. 6). Moreover, we conducted survival analysis to compare the recovery of potency between the 2 groups. The end-point event was set as patients achieving normal sexual function (IIEF-5 score > 21) or mild erectile dysfunction (IIEF-5 score = 12–21) after the surgery, which was constant with reference to our pre-surgical inclusion criteria. The results have been presented as Fig. 7, which shows that patients in the intrafascial group had faster recovery duration with a median survival time of 2.8 months when compared with that in the control group with a median survival time of 7.9 months. The Kaplan–Meier method was used, and statistically significant difference was set at *p* = 0.049. To furtherly affirm the effect of our modified intrafascial dissection on patients’ potency after surgeries, binary logistic model was also built to adjust interference of the confounding factors. We included patients’ age, BMI and pre-surgical IIEF-5 score in the equation as confounders. The results showed that after adjusting the effect of confounders, preservation technique could significantly influence the patients’ potency with a *p* value of 0.40. Odds ratio was 6.458 in favor of intrafascial dissection as compared with interfascial process.

**Table 5:** Comparison between completely intrafascial group and conventional interfascial group for erectile function

	Completely intrafascial group	Conventional interfascial group	*P* value
No. PDE5-Is prescribed (%)	14 (70.0)	18 (75.0)	0.711
Mean followed-up IIEF-5 score (SD)			
1 mo			
all	7.5 (3.6)	6.2 (2.9)	0.196
pre-operative normal EF	11.7 (0.6)	9.0 (2.9)	0.163
pre-operative mild ED	6.6 (3.4)	4.9 (1.9)	0.109
3 mo			
all	11.4 (3.5)	9.1 (3.2)	**0.026**
pre-operative normal EF	14.67 (0.6)	11.14 (2.7)	0.065
pre-operative mild ED	10.8 (3.5)	8.2 (3.0)	**0.027**
6 mo			
all	12.7 (3.3)	10.7 (3.8)	0.074
pre-operative normal EF	16.7 (3.1)	14.0 (3.7)	0.312
pre-operative mild ED	11.9 (2.8)	9.2 (2.8)	**0.009**
9 mo			
all	14.1 (3.6)	11.8 (3.3)	**0.039**
pre-operative normal EF	19.0 (1.0)	14.0 (3.6)	**0.049**
pre-operative mild ED	13.2 (3.2)	10.9 (2.7)	**0.035**
12 mo			
all	15.2 (3.8)	12.6 (3.6)	**0.033**
pre-operative normal EF	20.3 (0.6)	15.6 (3.7)	0.064
pre-operative mild ED	14.1 (3.2)	11.1 (2.6)	**0.011**
Mean change of IIEF-5 score from pre-surgical baseline (SD)			
1 mo			
all	−9.4 (3.8)	−11.1 (3.8)	0.161
pre-operative normal EF	−10.7 (0.6)	−13.7 (3.1)	0.146
pre-operative mild ED	−9.1 (4.1)	− 9.9 (3.6)	0.541
3 mo			
all	−4.9 (4.5)	−8.0 (4.4)	**0.026**
pre-operative normal EF	−7.7 (0.6)	−11.6 (3.0)	0.062
pre-operative mild ED	− 4.4 (4.7)	−6.5 (4.0)	0.168
6 mo			
all	−3.7 (3.7)	−6.6 (4.2)	**0.019**
pre-operative normal EF	−5.7 (3.2)	−8.7 (4.1)	0.291
pre-operative mild ED	−3.3 (3.8)	−5.7 (4.0)	0.086
9 mo			
all	−2.4 (3.5)	−5.4 (4.6)	**0.022**
pre-operative normal EF	−3.3 (1.2)	−8.7 (3.8)	**0.047**
pre-operative mild ED	− 2.2 (3.8)	−4.0 (4.3)	0.214
12 mo			
all	−1.7 (3.4)	−4.9 (4.4)	**0.016**
pre-operative normal EF	−2.0 (1.0)	−7.1 (3.9)	0.062
pre-operative mild ED	−1.6 (3.8)	−3.9 (4.3)	0.139

## Discussion

An increasing number of urologists have attempted to adopt preserving techniques in an attempt to improve patients’ post-operative functional outcomes after radical cystectomy. However, these modifications maybe compromise the oncological safety, leading to more local recurrences and metastatic diseases [6, 13]. Urothelial carcinoma should be considered as a potential lethal cancer, as relapse and metastasis can be catastrophic for patients and refractory for surgeons. Thus, privileging post-operative function at the cost of cancer relapse deserves to be carefully considered.

Mainly 3 types of techniques are applied to preserve the patients’ continence and potency following cystectomy: prostate/prostatic capsule-sparing technique, seminal-sparing technique, and nerve-sparing technique. Only limited evidences were available to evaluate the post-surgical effect when comparing these 3 techniques. A past retrospective analysis from a single-center demonstrated that these 3 techniques presented with comparable oncological outcomes in terms of local recurrence and cancer-specific survival, although capsule-sparing cystectomy and seminal-sparing cysto-prostatectomy showed a remarkably superior sexual function preservation rate when compared to that with nerve-sparing technique [14]. Similarly, in a randomized controlled study, Jacobs et al. identified a trend in favor of capsule-sparing cystectomy with regard to patients’ sexual function when compared with the nerve-sparing surgery, although no significant difference was tested (*p* = 0.06) [15]. However, the oncological risk of prostate/prostatic capsule-sparing and seminal-sparing surgeries was increasingly being questioned. Unlike the standard RC, transrectal biopsy or transurethral resection of prostate (TURP) was compulsively required before cystectomy [16, 17] so as to exclude prostatic-invaded urothelial carcinoma and prostate cancer. This pre-surgical approach may be bothersome and a financial burden for many patients. Moreover, TURP for patients with bladder cancer and sparing capsule and seminal vesicles may increase the risk of further metastatic diffusion [18]. Hautmann and Stein indicated that the distant failure rate of prostatic capsule- and seminal-sparing cystectomy was at least twice as high as that expected for a given series and > 5% higher as compared to that with the standard radical cystectomy [6].

Considering the oncological risk of prostate/prostatic capsule-sparing and seminal-sparing surgeries, we modified the conventional nerve-sparing technique and proposed our “completely intrafascial” cysprostatectomy. Present studies on male pelvic neuroanatomy have illustrated the pelvic plexus and the course of automatic nerves arising from it. At present, we have known that the pelvic plexus was located retroperitoneally on the lateral wall of the rectum with its midpoint related to the tip of the seminal vesicle [10]. Branches splitting from the pelvic plexus were arranged in a vertical plate lateral and closely adjacent to the seminal vesicles and then coursed to the prostate and surrounding the prostate [11]. The periprostatic nerves dispersed on the surfaces of the prostate and striated urethral sphincter in a cage-like fashion [19, 20]. Based on this understanding, we realized that the inferior effect of nerve-sparing technique to seminal-sparing surgery may be attributed to the injury of the nerves adjacent to the seminal vesicles and that the inferiority in preserving the capsule may be attributable to the removal of the nerves dispersing on the ventral and dorsal surfaces of the prostate. We modified the conventional maneuvers by dissecting the posterior bladder space internally to SVF and removing the prostate medially to the prostatic fascia, so as to protect and preserve the abutting nerves to an extreme. Puppo et al. reported a seminal-sparing technique combined with intrafascial prostatectomy to preserve the patients’ potency following cysprostatectomy [13]. The main difference from these techniques was that the entire seminal vesicles within SVF could be removed to preserve the adjacent nerves and thus reduced the risk of retaining tumor residue on the seminal vesicles.

Our retrospective study demonstrated that our proposed innovation is oncologically safe. Oncological outcomes did not differ in terms of the PSM and the recurrence and metastatic rates when compared to that with the conventional interfascial nerve-sparing surgery. This modified intrafascial cysprostatectomy led to a 10% relapse rate and a 10% metastatic rate within an average 15.7-months follow-up, which were comparable to those of the standard surgery. Moreover, all the patients receiving intrafascial techniques did not undergo any additional pre-surgical examination, either biopsy or TURP. Our previous systematic review confirmed the oncological safety of intrafascial prostatectomy with stringent case selection and patients’ inclusion [21]. Moreover, 2 cases of incidental prostate cancer were reported in this inferred group. The serum prostate-specific antigens of both the patients were < 0.1 ng/μL at 6-week after the surgery, and no biochemical recurrences were noted during the follow-up.

Included patients in our study had pre-operative clinical T1–3 stages bladder cancers and as shown in Tables 3, 3 cases and 5 cases were showed post-surgical pathological T4 stages in the intra- and inter-fascial group, respectively. Extravesical disease may compromise cancer control in the intrafascial group as our modified dissections were closer to the bladders. Evaluation systems based on preoperative imaging such as Vesical Imaging Reporting and Data System (VI-RADS) score could help asses bladder cancer staging before surgeries [22, 23]. Moreover, we included totally 6 cases with high-risk T1 stage cancers in our study. Prediction of high-risk was based on clinical-pathological parameters such as tumor grade, multifocality, size of tumors and the presence of carcinoma in situ. Some biomarkers such as baseline basophil count, survivin and circulating tumor cells could provide more accurate risk stratification to discriminate patients with higher risk T1 diseases who need early radical surgeries [24–26].

Regarding on patients’ post-surgical sexual function evaluated by IIEF-5 score, our retrospective data showed that completely intrafascial technique offered a significant advantage to preserve the patients’ post-surgical erectile function in comparison with the conventional nerve-sparing cystectomy, with higher means of the IIEF-5 score at 3, 9, and 12 months follow-up after cystectomy and a lower decrease from the baseline at 3, 6, 9, and 12 month follow-up after the surgery. Moreover, the survival analysis with the Kaplan–Meier methods indicated that the intrafascial approach offered a faster recovery of potency when compared to that with interfascial maneuvers. This could be explained by that our intrafascial dissection preserved more cavernous nerves adjacent to the seminal vesicles and dispersing on the surface of the prostates. Despite this effort, the nerve-sparing surgeries were still detrimental to patients’ potency. All the patients of both group revealed significant decreases of IIEF-5 score from baseline and especially at 1 month after surgeries, the mean decreases were − 9.4 and − 11.1 in intra- and interfascial group, respectively. These declines were improved to a large extent at 3 month follow-up because a big proportion of patients chose to take PDE5-Is due to unsatisfied intercourse. We stratified the included patients to normal EF and mild ED. It seemed that the IIEF-5 score of patients with normal erectile function before surgery descended more seriously as compared with patients with mild ED. Statistical significance could be detected in favor of intrafascial techniques in the mild ED subgroup in accordance with patients’ post-surgical IIEF-5 scores at 3, 6, 9, and 12 month follow-up. With regard to continence, intrafascial cystectomy seemed to offer a slight advantage for the earlier recovery of daytime and night-time urinary control, which was found to be constant with the one-arm consecutive series study conducted by Kessler [27]. However, the overall continence rate at 12 month failed to demonstrate any statistically significant difference between the 2 groups.

There were several limitations in our study. Firstly, it was a single-centered study. We reviewed the cases of last 5 years who were pre-operative potent and underwent nerve-sparing cystectomy. In fact, we had expanded the inclusion criteria by including patients with mild ED. But at last we only included overall 44 patients and the sample size was still insufficient. In the completely intrafascial group, only 3 patients had pre-operative normal sexual function and IIEF-5 scores greater than 21. Statistical significances could only be detected in a few comparisons maybe due to the insufficient cases in each subgroup. A main limitation of our study was the type of study design. It was a retrospective cohort study and the grade of evidence was low as compared with randomized controlled studies. From the year of 2007, we modified our technique and proposed the completely intrafascial cystectomy. Thus the follow-up duration of patients undergoing innovational cystectomy was shorter than the patients in control interfascial group. Regarding the inconsistency of follow-up, further verification is required to validate the present comparable oncological safety of intrafascial technique versus interfascial approach. Moreover, due to the short period of follow-up, we could not measure and compare the cancer specific survival and overall survival of patients between the two groups. At last, our results showed a significantly higher IIEF-5 scores and continent rates in favor of the modified technique, but the superiority was not dramatic, especially for the recovery of continence. Thus the advantages of our modified technique on patients’ sexual function and earlier recovery of continence needed further investigations by multi-centered, perspective and randomized trials in the future.

## Conclusions

We proposed a modified completely intrafascial cysprostatectomy to preserve the patients’ potency and continence. Our controlled study indicated that this technique provided superior erectile function and earlier recovery of continence for patients when compared with the conventional interfascial nerve-sparing approach. Moreover, completely intrafascial cysprostatectomy was oncologically safe and offered a comparable PSM and the recurrence and metastatic rates in comparison to that with the standard surgery. Considering the insufficient sample size and the limited follow-up, future studies with more number of centers are expected to investigate the long-term oncological and functional outcomes.

## Data Availability

The data used to support the findings of this study are available from the corresponding author upon request.

## Abbreviations

BMI: Body mass index
EF: Erectile function
DVC: Dorsal venous complex
IIEF: International Index of Erectile Function
MIBC: Muscle-invasive bladder cancer
PSM: Positive surgical margins
RC: Radical cysprostatectomy
SVF: Seminal vesicle fascia
TURP: Transurethral resection of prostate
